# Non-Pharmacological Therapy in Heart Failure and Management of Heart Failure in Special Populations—A Review

**DOI:** 10.3390/jcm13226993

**Published:** 2024-11-20

**Authors:** Jasmine K. Dugal, Arpinder S. Malhi, Noyan Ramazani, Brianna Yee, Michael V. DiCaro, KaChon Lei

**Affiliations:** 1 Department of Internal Medicine, University of Nevada Las Vegas, 1701 W. Charleston Blvd., Las Vegas, NV 89102, USA; jasmine.dugal@unlv.edu (J.K.D.); arpinder.malhi@unlv.edu (A.S.M.); noyan.ramazani@unlv.edu (N.R.); michael.dicaro@unlv.edu (M.V.D.); kachon.lei@unlv.edu (K.L.); 2 Division of Cardiovascular Medicine, University of Nevada Las Vegas, 1701 W. Charleston Blvd., Las Vegas, NV 89102, USA

**Keywords:** heart failure, lifestyle modification, diet, exercise, diabetes, chronic kidney disease

## Abstract

Non-pharmacological therapies play an essential role in the management of heart failure, complementing pharmacological treatments to mitigate disease progression and improve patient outcomes. This review provides an updated perspective on non-pharmacological interventions with a focus on lifestyle modifications, device therapies, and the management of heart failure in special populations, such as the elderly, women, and patients with comorbid conditions like renal dysfunction and diabetes. Key lifestyle interventions, including sodium and fluid restriction, dietary changes, and physical activity, are explored for their impact on symptom reduction, hospital readmissions, and quality of life. Device therapies like cardiac resynchronization therapy (CRT) and implantable cardioverter defibrillators (ICD) are also evaluated for their effectiveness in reducing mortality in patients with advanced HF. Special attention is given to vulnerable populations, emphasizing the need for individualized approaches tailored to specific pathophysiological mechanisms and socioeconomic factors. By integrating these strategies, healthcare providers can optimize care and enhance patient adherence, reducing the overall burden of heart failure.

## 1. Introduction

Heart failure (HF), a complex clinical syndrome characterized by the inability of the heart to pump blood effectively, remains a significant public health challenge, with increasing prevalence and associated morbidity and mortality. Specifically, HF refers to cardiac dysfunction linked to structural and/or functional incapabilities, either during ventricular filling or ventricular ejection [1]. In industrialized and high-income countries, the prevalence of heart failure (HF) is approximately 1–2% in the adult population, which increases to 10% when sub-stratifying the population of adults over 70 years of age [2]. Based on the guidelines of management of HF, the AHA/ACC/Heart Failure Society of America (HFSA) has classified HF into four main variations: heart failure with preserved ejection fraction (HFpEF), heart failure with reduced ejection fraction (HFrEF), heart failure with mildly reduced ejection fraction (HFmrEF), and heart failure with improved ejection fraction (HFimpEF) [1,2].

A guideline-directed medical therapy (GDMT) has been well studied and established as the mainstay of treatment. Yet, non-pharmacological management of HF is equally as important to mitigate and decrease the complications that arise from HF. However, poor compliance has been reported in the HF population, especially in the elderly, which results in an increased risk for mortality and HF readmissions [3]. The four core tenets of non-pharmacological management of HF include (1) adopting a lifestyle where patients follow a low sodium diet, (2) restricting the amount of fluid consumption, (3) weighing themselves daily, and (4) abiding with the recommendations and guidelines set for them by their physicians regarding exercise therapy and activity training [3]. Patients who were compliant with one modality (i.e., made modifications to diet and exercise) were also more eager and motivated to adopt secondary behavioral changes, such as decreasing consumption of alcohol and cigarette smoke [3]. This intrinsic behavioral modification among this class of patients caused a decrease in hospital readmission rates related to HF complications.

This review aims to provide an in-depth analysis of the current non-pharmacological interventions for HF, focusing on their effectiveness in diverse populations. Special attention will be given to particularly vulnerable groups, such as the elderly, women during pregnancy, and individuals with concomitant renal dysfunction or diabetes. Moreover, this review will explore the impact of HF across different socioeconomic and financial statuses and between various population classes, racial backgrounds, and age groups. This manuscript provides a more comprehensive approach to the non-pharmacological management of HF that encompasses the many facets of preventative measures and treatment regimens and discusses the importance of compliance and adherence to such therapies and interventions.

## 2. Lifestyle Modifications

Effective lifestyle modifications are an integral component in the management of HF, complementing GDMT by reducing symptom burden and hospitalizations and concomitantly improving quality of life. The interplay between cardiac, renal, and endocrine systems, termed cardio–kidney–metabolic (CKM) syndrome, requires a multifaceted approach to HF treatment. The major tenets of lifestyle modification include sodium restriction, fluid restriction, dietary adjustments, physical activity, and weight management.

### 2.1. Sodium Restriction

The mechanism of sodium restriction improvement has been a subject of debate, particularly regarding sodium stimulation of the neurohormonal system. Increased salt retention by kidneys leads to worsening edema and congestion and stimulates increased sympathetic activity. This results in peripheral vasoconstriction of the afferent renal artery and decreased blood flow to the juxtaglomerular apparatus in the kidneys, thereby activating the renin–angiotensin–aldosterone system (RAAS). Angiotensin II (ATII) directly causes sodium retention in the proximal tubules, while aldosterone leads to increased sodium resorption in the distal tubule and plays a key role in worsening heart failure [4].

It has also been shown that water retention and volume overload in acute heart failure exacerbation are primarily driven by sodium retention [5]. However, experts have suggested sodium restriction in patients with poor nutritional status may lead to poorer results in heart failure patients due to reduced appetite and food intake, exacerbating malnutrition. In fact, a systematic review and meta-analysis of 10 randomized controlled trials were performed (1011 participants with HF) to evaluate the effects of dietary sodium restriction on quality of life (QoL), and the results showed that sodium restriction did not improve QoL over long term (>30 days) (*p* = 0.61). The pooled results also showed that sodium restriction might increase mortality risk (*p* < 0.00001). Lastly, it also showed that it did not reduce the readmission rate within the short term (≤30 days) (*p* = 0.78) and, on the contrary, increased the readmission rate over the long term (*p* = 0.0003) [6]. Despite its controversy, sodium restriction has been internationally acknowledged as a method of lifestyle modification in HF patients.

Dietary sodium restriction in heart failure patients has been recommended by many different guidelines. The Korean Society of Heart Failure (KSHF), European Society of Cardiology (ESC), and American Heart Association/American College of Cardiology (AHA/ACC) all recommend sodium restriction. However, the consensus among the societies varies. The Korean heart failure guidelines recommend sodium restriction to <2 g per day in moderate to severe heart failure [7], while European guidelines recommend avoiding excessive sodium (>5 g/day) [8]. Per AHA/ACC guidelines, patients should avoid excessive sodium to reduce congestive symptoms [9]. Even though guidelines have recommendations regarding sodium restriction in HF patients, there is a disagreement among experts about sodium restriction, especially in malnourished patients. Because of the variability in recommendations and patient responses, an individualized approach to sodium restriction, particularly in patients with comorbid conditions or malnutrition risk, may be more beneficial than a one-size-fits-all approach.

### 2.2. Fluid Restriction

The benefit of fluid restriction in HF patients is similarly a subject of debate, with inconsistent evidence supporting its routine use [5]. A small single blind study was carried out by Travers et al. to investigate the clinical impact of fluid restriction in class IV heart failure patients during their hospitalization. The study showed no significant difference in time to clinical stability, discontinuation of IV diuretic therapy, or stabilization of serum urea, serum creatinine, natriuretic peptides, or sodium in fluid-restricted patients (*n* = 34) versus free-fluid patients (*n* = 33) [10]. A key study examining the outcomes with 1 L fluid restriction in patients found that quality of life improved, though it did not find any differences in HF rehospitalization or all-cause mortality [11]. Given the limited data on fluid restriction, new randomized control studies are needed to adequately determine the utility and effectiveness of fluid restriction.

Subsequently, there are no clear recommendations in clinical guidelines for fluid restriction. KSHF guidelines suggest educating patients about fluid restriction at discharge following a hospitalization for heart failure [7]. However, KSHF does not specify an exact amount of fluid restriction in the guidelines. Conversely, ESC guidelines recommend considering a fluid restriction of 1.5 to 2 L in patients with severe heart failure/hyponatremia to relieve symptoms and congestion, taking note to increase fluid intake during periods of high heat/humidity and/or nausea/vomiting [8]. AHA/ACC guidelines make a 2b recommendation regarding fluid restriction in heart failure patients as the benefit of fluid restriction to reduce congestive symptoms is uncertain [9]. Ultimately, fluid restriction should be considered on a case-by-case basis, with careful attention to patient-specific factors such as comorbid conditions and symptomatology.

### 2.3. Dietary Changes

Dietary interventions are essential in managing heart failure, particularly in patients with comorbid obesity or metabolic syndrome. A healthy diet should be adapted to the specific needs of each patient. Nutrition with restricted caloric intake designed for weight loss is recommended in overweight or obese HF patients. Patients who are overweight or obese have a significantly increased risk of developing HF, suffering from more frequent HF exacerbations, and experiencing progression of HF. Particularly, the increased cardiac work required to maintain cardiac output in such individuals increases ventricular strain, leading to earlier onset and worsening of HF [12]. The newly termed CKM syndrome has emerging evidence on the risk of cardiovascular disease (CVD), including HF [9,13]. Risk calculators such as PREVENT for the staging of CKM may help providers evaluate an individual’s risk for the development of CVD and guide management accordingly. This model includes considerations of sex, age, comorbidities such as diabetes and CKD, and lifestyle habits including tobacco use [13].

While neither the KSHF nor the ESC provides specific dietary recommendations, the AHA/ACC guidelines recommend adherence to heart-healthy, evidence-based diets such as the Dietary Approaches to Stop Hypertension (DASH) diet and the Mediterranean diet. The DASH diet focuses on reducing sodium intake and increasing consumption of fruits, vegetables, and low-fat dairy products. The DASH diet, through the original DASH trial, was shown to substantially reduce blood pressure compared with a diet that is low in these foods [14,15]. Furthermore, consistent compliance with diets that are low in sodium and high in fruits, vegetables, low-fat dairy products, antioxidants, and potassium may be linked to decreased heart failure hospitalizations [16]. The Mediterranean diet, a generic term used to describe a dietary pattern consisting of fruits, vegetables, unsalted nuts, whole grains, lean proteins, and extra-virgin olive oil, has been shown through multiple studies to reduce blood pressure and improve overall cardiovascular health [17,18].

Elderly HF patients, particularly those who are frail, often face barriers to maintaining a heart-healthy diet as they are more likely to be dependent on caregivers for meal preparation. A study of 40 patients found that both patients and caregivers consume poor-quality diets, which suggests that even though the Mediterranean diet and DASH diet have beneficial effects, the focus should be placed on household modifications for heart failure patients to have any success [9].

In patients with advanced HF (AHA/ACC stage C and D), unintentional weight loss, sarcopenia, and cardiac cachexia are common, which further complicates the management of nutritional intake. These conditions are associated with lower caloric intake and higher micronutrient deficiencies compared with age- and sex-matched healthy adults [19]. Additional weight loss to address obesity may exacerbate these conditions. Dietary changes need professional guidance and counseling on adapting a diet to reduce weight loss that is driven by malnutrition to avoid sarcopenia and cardiac cachexia. Strategic treatment for sarcopenia includes resistance training exercises to improve overall muscle strength and mass with focused nutritional support in protein intake. Cardiac cachexia is a complex metabolic syndrome that can be addressed through high-calorie, high-protein diets. At times, appetite stimulants may also be required, especially in end-stage heart failure. Both sarcopenia and cardiac cachexia are factors associated with worsened prognosis in heart failure [20].

### 2.4. Physical Activity

Physical activity has been linked to increased quality of life, exercise capacity, and decreased hospitalizations. Exercise-based cardiac rehabilitation programs have been shown to significantly enhance functional status, as measured by the 6 min walk test and reduce all-cause and HF-related hospitalizations [21]. Such classes have tailored exercises for cardiac patients to improve heart health. Current guidelines strongly recommend cardiac rehabilitation programs (CRP) for all eligible heart failure patients though practical data show low participation rates [21]. A CRP is primarily based on physical exercise; however, a comprehensive CRP also includes educational sessions that focus on risk factors, lifestyle modification, nutritional advice, psychosocial support, smoking cessation, regulation of body weight, and optimization of blood pressure, lipid levels, and glycemic control [22]. General exercise and physical activity are still highly recommended, particularly for patients who do not qualify for CRP or where CRP is not available. Patients who have completed CRP are also recommended to continue physical activity, utilizing learned skills for future habits. Guidelines recommend exercise as a non-pharmacological treatment for heart failure but do not specify intensity or duration. KSHF guidelines recommend promoting exercise-related activities, along with an exercise prescriber, in a multidisciplinary team that handles heart failure patients [7]. ESC recommends exercise for all patients who are capable of doing so [8]. The 2022 AHA/ACC Heart Failure Guidelines also recommend exercise training or regular physical activity to improve functional status, exercise performance, and quality of life in heart failure patients who can participate in the exercise training [9].

### 2.5. Weight Management

Obesity can lead to heart failure directly (through the effects on the myocardium) and indirectly (through obesity-related comorbidities). Obesity can lead to hemodynamic changes, including increased blood volume and cardiac output through activating RAAS. Apart from hypertension, it can also increase the risk of diabetes and hyperlipidemia, all of which are risk factors for developing heart failure. Obesity can also directly affect the heart through fat accumulation in the myocardium, which can lead to fibrosis and the development of heart failure [23]. In fact, a study performed by Kenchaiah et al. investigated the relation between body mass index (BMI) and the incidence of heart failure among 5881 participants and found that after adjustment for established risk factors, there was an increase in the risk of heart failure of 5 percent for men and 7 percent for women for each increment of 1 in BMI increase. Compared with participants with normal BMI, obese participants had a doubling of the risk of developing heart failure [24].

Weight loss is recommended to help prevent heart failure; however, in patients with established heart failure, the efficacy of weight loss is less clear [5]. Even though obesity increases the risk of heart failure, it has a protective effect in patients already diagnosed with heart failure as a lower body mass index (BMI) is associated with a higher risk of mortality. This phenomenon has been called the “obesity paradox”. The obesity paradox is typically observed in mild obesity and seems to lose any potential survival benefit in severely obese patients [25].

Weight loss has been shown to reduce the incidence of heart failure, but in patients with known heart failure, unintentional weight loss can be detrimental. This even includes patients with mild heart failure as weight loss >5% over one year was a significant predictor of cardiovascular death or hospitalization with heart failure exacerbation [26]. The 2016 ESC guidelines stated that in patients with heart failure and moderate obesity (BMI <35 kg/m^2^), weight loss cannot be recommended. Patients with a BMI of 35–45 kg/m^2^ may consider weight loss for symptom management and exercise capacity [25]. The ACC/AHA guidelines also have a class 1 recommendation stating that conditions that may lead to or contribute to heart failure, such as obesity, should be controlled [25]. However, due to the paucity of data, firm guidance regarding the management of obesity in heart failure patients is lacking.

### 2.6. Substance Use Cessation

**Figure 1:**
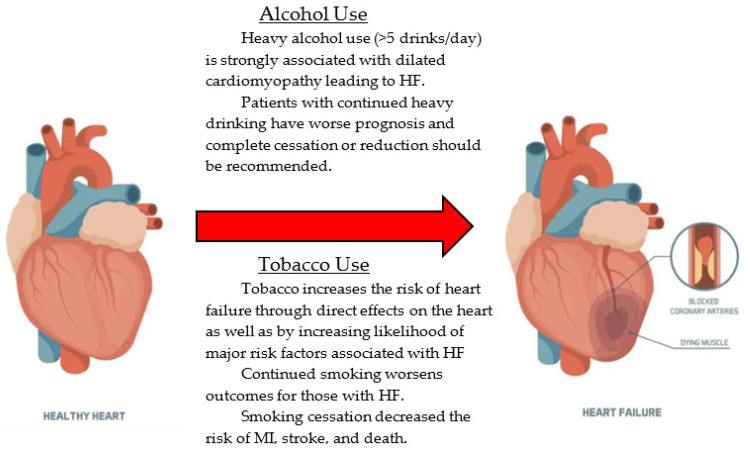
The effects of alcohol and tobacco on the heart. Heart failure (HF). Myocardial infarction (MI).

Substance use, particularly alcohol, tobacco, cannabis, and cocaine, is strongly linked to the onset and progression of HF. Moderate alcohol may reduce the incidence of heart failure, but heavy alcohol usage (>5 drinks per day) is strongly associated with dilated cardiomyopathy [27]. In addition, tobacco usage also increases the risk of heart failure by increasing the risk of coronary artery disease (CAD), along with other comorbidities that contribute to CAD, including hypertension, diabetes mellitus, and hyperlipidemia [5,28]. Smoking has a direct effect on the heart as the chemicals that are inhaled damage the blood vessels and form plaque in the coronary arteries, leading to CAD. Figure 1 below summarizes the effects of smoking and alcohol as well as the benefits of smoking and alcohol cessation.

Patients who engage in heavy drinking or smoking should be strongly advised to stay away from heavy and binge drinking [5]. Specifically, smoking cessation has been shown to lower the incidence of serious adverse events, such as myocardial infarction, heart failure, stroke, and death [27]. Healthcare providers should emphasize the importance of substance use cessation as part of comprehensive lifestyle modification strategies for HF patients.

### 2.7. Weight Monitoring and Education

Regular weight monitoring in patients is critical, particularly in detecting fluid retention, which may indicate worsening heart failure. The WHARF trial, a large randomized controlled study, investigated patients with NYHA class III or IV heart failure and LVEF ≤ 35%. It compared heart failure outcomes between those receiving standard care and those receiving standard care alongside access to the AlereNet System, a technology-based monitoring system designed to track daily weight and symptoms in heart failure patients. The results revealed a 56.2% reduction in mortality (*p* < 0.003) for patients in the AlereNet group [29].

A sudden increase in weight often indicates fluid overload, which can occur even in patients compliant with treatment regimens and lifestyle modifications [9]. Accordingly, patients are advised that an increase of two to three pounds per day, or five pounds in one week, will require adjustment to their medication regimen. Recognition of these bodily changes empowers patients to engage in their care, adjust lifestyle factors, and recognize when to seek prompt medical advice to reduce hospital admissions for heart failure exacerbations.

To ensure the accuracy of the daily weight monitoring, providers should educate patients to weigh themselves at the same time every morning, preferably after using the restroom and before eating or drinking [9]. Patients are advised to use the same scale and wear similar clothing daily. These daily weights should be logged, and any concerning trends should be promptly discussed with the healthcare provider. This allows patients to play an active role in managing their heart failure condition, leading to improved outcomes and fewer heart failure exacerbations [9].

## 3. Advance Interventions: Devices and Surgery

In addition to lifestyle modifications, device therapy, and surgery present another avenue of evidence-based non-pharmacological management of heart failure, especially in severely reduced HF and advanced HF. Cardiac resynchronization therapy (CRT) and implantable cardioverter defibrillators (ICD) are viable options for helping patients with HF reduce overall mortality and prevent sudden cardiac death (SCD).

CRT assists with coordinating cardiac contractions in the lower chambers of the heart and has also been found to decrease the incidence of life-threatening arrhythmias like ventricular tachycardia (V-Tach) and ventricular fibrillation [30]. Relevant indications for CRT include HFrEF with LVEF ≤ 35%, symptomatic heart failure NYHA class II, II, or ambulatory IV despite optimal medical therapy, and prolonged QRS ≥ 130 milliseconds (Table 1) [31].CRT is a viable option for treatment in HF patients who have a conduction system abnormality. Even with a substantial non-responder rate of 30%, CRT can lengthen the time to heart transplantation and left ventricular assist device placement in patients with HF [32]. The MIRACLE Trial of 2002 showed the following improvements for patients on CRT versus traditional control groups on standard conventional therapy with heart failure: improved distance walked in six minutes, decreased need for peak oxygen consumption, and improved NYHA class [33].

ICD, which can be an additional feature of CRT, is a device utilized to monitor the heart rhythm and deliver a coordinated shock to prevent SCD caused only by ventricular tachyarrhythmia, severe bradycardia, or complete heart block [34]. HF patients with indication for ICDs include those with LVEF ≤ 35%, symptomatic HF, history of V-fib or sustained V-tach, recent MI within last 40 days with LVEF ≤ 35% and symptomatic HF, nonischemic cardiomyopathy and LVEF ≤ 35%, and long-standing symptomatic HF despite optimal medical therapy (Table 1) [35]. An association was found between reduced all-cause mortality when substratified for age, such that patients who were <70 years of age and had nonischemic systolic heart failure benefited from ICD implantation [27]. In a study on long-term follow-up, patients in the DANISH trial showed that the ICD group had a lower incidence of SCD. This impact was more pronounced in patients <70 years of age, but not in the group of patients >70 [36].

Potential complications or risks associated with device usage include perioperative complications such as pneumothorax, bleeding, and cardiac perforation, and late complications associated with ICD treatment such as inappropriate shocks, device-related infection, the fear of appropriate shocks, and quality of life [34]. Key aspects of CRT and ICD therapies are discussed in Table 1 below.

In patients diagnosed with NYHA functional classes II–IV secondary to mitral regurgitation, MitraClip implantation lowered 2-year mortality rates or heart failure-related hospitalizations compared with only providing GDMT. MitraClip valve repair also improved quality of life at two years compared with GDMT alone, independent of baseline functional status [37].

Ischemic cardiomyopathy leading to HfrEF may be reversible and can improve with revascularization. Evidence surrounding PCI versus coronary artery bypass grafting (CABG) has shown that CABG offers substantial survival benefits and significant reduction in myocardial infarction and the need for repeat revascularization in multivessel CAD (MVCAD), particularly in diabetic patients with intermediate and high severity disease [38]. Additionally, patients who underwent coronary artery bypass grafting (CABG) surgery had substantially less risk of mortality from any cardiovascular cause compared with those only receiving traditional medical therapy [39].

## 4. HF Management in Special Populations

Special populations within HF, such as the elderly, women, and patients with comorbid conditions, face unique challenges that require tailored strategies to optimize outcomes. Understanding the personalized challenges within and between groups allows providers to connect with their patients on an individual level and provide precise, personalized care. This section highlights the fundamental mechanisms, management strategies, and population-specific considerations to aid providers in treating special populations of people with HF. Important takeaways and key considerations for HF management in special populations are summarized in Table 2.

### 4.1. Heart Failure in Elderly Patients

#### 4.1.1. Mechanism of Heart Failure in Elderly Patients

Aging is a normal process that leads to decreased cardioprotective systems and increased disease processes that may lead to heart failure [40]. Even though aging does not cause heart failure, it lowers the threshold for disease manifestation [40]. The elderly population has an increased prevalence of comorbid conditions that increase cardiovascular risk, such as hypertension, hyperlipidemia, and coronary artery disease, resulting in increased risk for heart failure. Older patients with HF also experience a high burden of polypharmacy, frailty, and cognitive impairment [41]. Changes in arterial vessels and consequences with advanced age involve increased oxidative stress, endothelial dysfunction, inflammation, matrix metalloproteases, and vascular cell proliferation [40]. This leads to changes in arterial walls through decreased elastin content, increased collagen production, cross-linking and glycosylation, decreased collagen degradation, and increased intima medial wall thickness [40]. The hemodynamic consequences of this change result in increased diameter of central arteries, systolic pressure, pulse pressure, LV afterload, and oxygen requirements, as well as a decrease in coronary filling pressures and LV early diastolic filling [40]. Table 3 below provides a summary of age-related changes.

#### 4.1.2. Management Considerations in the Elderly Population

Noncompliance proves to be a large barrier to improvement for HF patients, and the elderly population remains especially vulnerable due to financial difficulties, social isolation, and cognitive impairments that preclude complex medication regimens, among others [40]. Older individuals who are diagnosed with HF have similar recommendations for GDMT to the general population; however, each of these medications has specific, unique mechanistic effects that can be utilized to the advantage of the patients and their comorbidities.

Given that the elderly population is more sensitive to small changes in intravascular volume, diuretics have been proven effective in reducing congestive symptoms [40]. The ultimate effect of beta blockers is to inhibit the sympathetic nervous system, which is often upregulated in heart failure. By reducing heart rate and myocardial oxygen demand, beta-blockers improve left ventricular function and reduce mortality and hospitalization rates [9]. MRAs block the effects of aldosterone, thereby reducing sodium retention, myocardial fibrosis, and ventricular remodeling, leading to improved survival and reduced hospitalizations [42]. ACEis, ARBs, and ARNIs reduce the detrimental effects of RAAS activation, thereby reducing vasoconstriction, sodium retention, and myocardial fibrosis, consequently decreasing afterload and reducing ventricular remodeling [9]. HF in elderly patients is associated with increased activation of the sympathetic system as well as the RAAS; as such, beta-blocker therapy and ACE inhibitor therapy are helpful in the elderly population. Elderly patients tend to exhibit more sensitivity to GDMT, and careful titration of GDMT should be considered to avoid significant hypotension and/or bradycardia.

Exercise training can also be generalized to older patients, as the disease process is associated with decreased muscle mass and strength. Although advances in pharmacological therapies available for heart failure exist, up to 40% of patients with HF will die within one year of their first hospitalization [41]. We emphasize the importance of rigorous patient education, close monitoring, and social support to help reduce the disparity.

### 4.2. Heart Failure in Females

Heart failure phenotypes can differ based on sex. HfpEF is more prevalent among females, and HfrEF is higher among males [43]. Hypertension (HTN) and diabetes contribute to the risk for HF in females, whereas ischemia is the leading cause of HF in males [44]. Sudden cardiac death, which can be caused by HF, is also more common in males compared with female counterparts, primarily attributed to the fatal arrhythmias associated with HfrEF that may not be seen in HfpEF [45].

#### 4.2.1. Mechanism of Heart Failure in Females

Over the last decade, the hallmark of HfpEF in females has been described by the increased stiffness and smaller size of the left ventricle compared with males. Consequently, females require a greater resting heart rate than males to maintain cardiac output. With HfpEF and aging, an overall increased stiffness of the LV and stroke volume reduction occur, leading to dependence on increased heart rates to compensate for cardiac output.

Although less prevalent, women with HfrEF may present with increased morbidity compared with their male counterparts. One study found that women with severe HfrEF had lower exercise tolerance, worse pulmonary function, and worse kidney function than males of similar age and ejection fraction [44]. The PREVENT model for HF incorporates sex into consideration of the overall risk for HF development.

#### 4.2.2. Females with Comorbid Diabetes

Growing evidence indicates that women with diabetes experience more pronounced endothelial, coronary microvascular, and diastolic abnormalities than men with diabetes. The exact mechanisms behind the heightened risk of heart failure in diabetic women remain unclear. Still, factors such as sex hormones, variations in cardiovascular risk profiles, and potential differences in treatment patterns between men and women might contribute [46].

#### 4.2.3. Pregnancy and Heart Failure

Pregnancy has a known association with peripartum cardiomyopathy (PPCM) and can be a life-threatening syndrome in women. As pregnant patients near the end of pregnancy and in the weeks to months after delivery, left ventricular systolic dysfunction is observed and leads to an increased risk for heart failure. The potential etiology includes inflammation and angiogenic dysregulation, causing vascular damage. Patients usually present with an ejection fraction (EF) of <45% [44].

#### 4.2.4. Management Considerations for Female Patients

There is not much known regarding the sex-specific aspects in the prevention or treatment of HF; however, a prospective study that followed adults with five types of dietary patterns noticed that adherence to a plant-based dietary pattern, more frequently found in women, was inversely associated with incident HF risk. In contrast, the Southern dietary pattern was positively associated with incident HF risk [47]. Obesity, anemia, and depression are more prevalent in females with heart failure, and as such, treating these conditions effectively can help manage heart failure. Current research has not found a benefit to estrogen therapy though targeting hormonal changes during and after menopause may also be considered in the future. Although the loss of estrogen is linked to an increased cardiovascular risk, hormone replacement therapy (HRT) is generally not recommended in women with heart failure due to concerns about clotting and worsening heart failure.

#### 4.2.5. Management Consideration for Pregnant Females

Management of heart failure in pregnancy aims to relieve symptoms, optimize hemodynamic status, improve long-term outcomes with continuation or initiation of therapies for chronic conditions, and treat other precipitating factors such as anemia, thyroid imbalance, infections, and arrhythmias [48]. Chronic heart failure in pregnancy warrants lifestyle modifications, vaccinations, and, possibly, even device therapy such as cardiac resynchronization and/or implantable cardioverter defibrillator therapy [48]. Non-pharmacological management of acute and refractory heart failure in pregnancy includes supplemental oxygen therapy and temporary mechanical circulatory support [46].

### 4.3. Heart Failure in Renal Dysfunction

#### 4.3.1. Mechanism of Heart Failure in Renal Dysfunction

Heart failure can exacerbate renal dysfunction and vice versa, as third-spacing of fluid in congestive heart failure (CHF) can induce acute kidney injury (AKI), leading to further depletion of intravascular volume. The Acute Decompensated Heart Failure National Registry (ADHERE) calculated that approximately one-third of all patients who have been hospitalized in the past for acute decompensated HF have acute or chronic renal insufficiency [49]. The classic interplay between heart failure and kidney disease, termed cardio-renal syndrome, has three significant comprehensive interactions: (1) hemodynamic mechanisms, (2) neurohormonal mechanisms, and (3) CVD-associated mechanisms [49].

Due to low systemic perfusion rates as a consequence of acute HF exacerbation, the nephron can temporarily avoid damage by activating the RAAS pathway initiated by the glomeruli in this reactive state. This temporary sub-optimal state will eventually lead to exhaustion and depletion of homeostasis and to overt kidney damage, surpassing a simple AKI. HF with ‘forward failure,’ or a drastic decrease in cardiac output due to HfrEF, can lead to renal tubule hypoxia and acute tubular necrosis [49]. Continuous and recurring insult to the kidneys leads to the development of chronic kidney disease (CKD), severe electrolyte derangements, rise in creatinine and blood urea nitrogen levels (BUN), and elevation of other chemical and enzymatic biomarkers in the blood, causing an overall toxic environment.

#### 4.3.2. Management Strategies for Renal Dysfunction

The specific management strategies of renal dysfunction in the setting of HF encompass both a non-pharmacological device-driven therapeutic approach and a pharmacological approach. The value of cardiac resynchronization therapy (CRT) as an intervention in patients with an eGFR < 60 mL/min has been just as advantageous and efficacious as in patients with an eGFR > 60 mL/min [50]. As such, the usage of CRT, regardless of eGFR in CKD patients, may have equal benefits for reduction in death and HF hospitalizations. Accordingly, CRT-D therapy demonstrated a significantly lower risk of mortality and heart failure hospitalizations compared with ICD [51].

Placement of an ICD as a primary prevention against sudden cardiac death (SCD) may prove to be beneficial for patients with CKD stage 3 and concomitant HfrEF [52]. The risk for SCD in patients with advanced CKD or dialysis is higher than either risk factor alone [53]. However, recommendations remain to implant ICDs in patients with EF < 35% as risk for infection in dialysis patients proves to be a significant consideration.

Pharmacological interventions in patients with HfrEF and CKD are vast and include standard GDMT, with a focus on angiotensin-converting enzyme inhibitors (ACEi), angiotensin receptor blockers (ARBs), hyperpolarization-activated cyclic nucleotide-gated (HCN) channel blockers like Ivabradine, angiotensin receptor/neprilysin inhibitors (ARNI), and sodium-glucose co-transporter-2 (SGLT-2) inhibitors. For African American patients with NYHA class III-IV HfrEF, hydralazine and isosorbide dinitrate reduce morbidity and mortality [54].

Targeting the RAAS pathway with ACEi, ARB, and ARNIs has been most utilized in patients with CKD given its mechanistic effects, as explained previously. GDMT initiation becomes most effective when proactively planning the regimen based on future transitions to other classes. Transitions between two classes of medications, such as ARBs, then transitioning to ARNIs is preferred to transitioning from ACEi to ARNIs due to the presence of a 36 h washout period that is needed for this particular transition. The high prevalence of side effects such as angioedema and cough are common complaints among patients who are on ACEi, which also makes ARBs/ARNIs more optimal. However, landmark trials do not establish a specific order of GDMT administration that is preferred over another.

SGLT2is, initially developed for diabetes, reduces HF hospitalizations and cardiovascular mortality through mechanisms such as natriuresis and reduction in preload and afterload [42]. Newly emerging data from the SCORED trial have linked improvement in mortality and heart failure hospitalizations with sotagliflozin, which is a nonselective SGLT inhibitor [55]. Though not implemented in guidelines yet, we highlight this as a potential consideration for patients with CKD as a possible medication during early GDMT titration.

Ivabradine, another GDMT option, selectively inhibits I_f_ (funny) channels in a concentration-dependent manner, reducing HR [56]. Ivabradine has a selective advantage in CKD patients as its clearance through the renal tubules only contributes to 20% of the total metabolism of the drug [56]. Careful initiation must be considered for patients with concomitant liver conditions as serum concentration of ivabradine can be increased in chronic liver and cirrhosis patients. Research has shown that patients with a Child–Pugh score ≤7 can have increased concentration of ivabradine levels, but generally, there are limited data on studies with patients diagnosed with moderate or severe hepatic impairment [56].

### 4.4. Heart Failure in Diabetes

#### 4.4.1. Mechanism of Heart Failure in Diabetes

Diabetic patients have over twice the risk of developing HF than patients without diabetes. Diabetes mellitus is associated with increased myocardial fatty acid utilization, decreased glucose utilization (glycolysis and glucose oxidation), increased myocardial oxygen consumption, and decreased cardiac efficiency. In diabetes, fatty acid oxidation occurs at an increased rate, but triglycerides and other lipid metabolites tend to accumulate in the diabetic heart. It is suggested that diabetes increases the risk of heart failure up to twofold in males and fivefold in females. This increased incidence persists despite adjusting for risk factors such as age, HTN, HLD, and CAD [57].

#### 4.4.2. Management Considerations in Diabetes

An important aspect of heart failure management with concurrent diabetes consists of dietary and lifestyle modifications, which are similar to the lifestyle modifications discussed above. In general, patients are recommended to take a multi-lifestyle approach through the “Life’s Essential 8” [58]. The ideal Life’s Essential eight components include nonsmoking, body mass index <25 kg/m^2^, ideal physical activity, ideal diet score, serum cholesterol <200 mg/dL without medication, blood pressure <120/<80 mmHg without medication, sleep health, and fasting glucose <100 mg/dL without medication [58,59].

Nutrition plays a vital role in HF and diabetes; however, dietary plans must be tailored according to personal and cultural food preferences, required caloric intake, comorbidities, current medications, and need for weight loss. Regardless, for those with diabetes and HF, minimizing alcohol intake and avoidance of smoking are always recommended [46].

HF is associated with physical inactivity and low fitness; given the high prevalence of concomitant HF and diabetes, exercise therapy can and should be directed to this population as well. Cardiac stiffness, the mainstay of HfpEF, accelerates in midlife but can be reversed by aerobic exercise [46]. One study consisting of 2331 patients, 32% with diabetes, was randomized to an aerobic exercise training program or standard of care for HF and then followed for 2.5 years. It was noted that although all individuals had a baseline lower functional capacity at the start of the study, those who completed the exercise program had significant improvement in their peak oxygen consumption and distance covered in the six-minute walk test compared with those who received only the standard care without any exercise training. The patients enrolled in this study who also had diabetes accounted for a large portion of the sample and, therefore, support the recommendations for exercise therapy to improve functional capacity in both heart failure and diabetes alike [60].

Several agents that demonstrate cardiovascular safety and improved outcomes also serve as effective antihyperglycemic agents, specifically those studied in heart failure patients. These include dipeptidyl peptidase-4 (DPP-4) inhibitors, glucagon-like peptide-1 (GLP-1) agonists, and sodium-glucose cotransporter-2 (SGLT2) inhibitors [61]. DPP-4 inhibitors increase the concentration of GLP-1. Small human studies with GLP-1 agonists exhibited similar results, with an overall increase in LVEF and functional status noted within the participants [62]. GLP-1 agonists have shown promise in HF treatment not only due to their beneficial effects on cardiovascular outcomes but also by managing other organ systems that relate to HF morbidity, including targeting weight loss and glycemic control, which work through the reduction of metabolic complications associated with obesity and HF, MACE, and HF symptoms [63]. The multisystem approach through GLP-1 agonists has led to a new topic of discussion about its effectiveness in HF, along with other medications for diabetes management. SGLT2 inhibitors have been shown to improve blood pressure control, arterial stiffness, vascular resistance, and microvascular remodeling—all occurring via multiple proposed mechanisms, including the beneficial effects on ion homeostasis, calcium handling, reduction of cardiac oxidative stress, and vascular inflammation. Myocardial metabolism utilizes ketone bodies as an energy-efficient source instead of glucose and lipid oxidation, which leads to improved cardiac contractility and confers cardiovascular protection.

**Figure 2:**
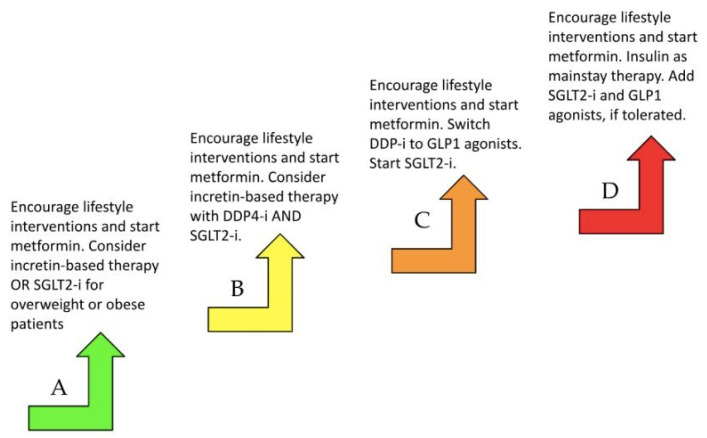
The recommendations for antihyperglycemic therapy based on the heart failure stage. Sodium-glucose cotransporter-2 (SGLT2). Glucagon-like peptide-1 (GLP-1). Dipeptidyl peptidase-4 (DPP-4).

As SGLT2i has been incorporated into GDMT, patients with diabetes are recommended to start empagliflozin or dapagliflozin earlier rather than later for optimal benefits. The DAPA-HF trial found that the risk of worsening heart failure or death from cardiovascular etiologies was lower in patients on dapagliflozin therapy compared with those who received placebo, regardless of the presence or absence of diabetes [64]. Another relevant trial that proved the cardiac benefits of early SGLT2-i is the EMPEROR-preserved trial, which showed that empagliflozin reduced the combined risk of cardiovascular death or hospitalization for patients with HFpEF [65]. Incretin-based therapies such as DPP-4 inhibitors and GLP-1 agonists should also be prioritized in diabetes management in these patients, possibly adjusting patients’ diabetes regimen to include either class. Figure 2 below denotes recommendations for stepwise antihyperglycemic therapy based on heart failure stages A through D, indicated at each arrow [61].

Briefly mentioned above, nonselective SGLT inhibitors may also play a role in diabetes patients with HF. The SOLOIST-WHF trial, which enrolled patients with diabetes and recent worsening heart failure, noted that sotagliflozin (SGLT1 and 2 inhibitors) therapy significantly lowered total number of deaths from cardiovascular causes and decreased the number of hospitalizations and urgent visits for heart failure compared with placebo when the medication was initiated before or shortly after discharge [55].

### 4.5. Ethnic and Racial Minorities and Heart Failure

Broadly, HF prevalence varies depending on the racial communities; African Americans have a higher hazard ratio of HF, followed by Latin X/Latinos, Caucasians, and Asian communities [66]. HFpEF is more prevalent in non-African American demographics [43]. Comorbidities appear to primarily affect the presence of HF. African Americans exhibit a higher burden of hypertension (HTN), and Hispanics have more complications of diabetes mellitus (DM), both of which are increased risk factors for HF [66]. Thus, the management and prevention of HF are rooted in managing comorbidities, as addressed in previous sections.

### 4.6. Social Determinants of Health (SDOH), Socioeconomics, and Heart Failure

HF patients experiencing SDOH, particularly those from low-income or underserved populations, face numerous barriers to implementing non-pharmacological therapies for HF management. These barriers are primarily related to a lack of access to care in the form of cost and transportation issues, which increases the likelihood of poor HF outcomes over time due to more frequent symptom exacerbations and recurrent hospital admissions [66]. A significant challenge for this population is the cost associated with managing complex chronic illnesses, including medication and healthcare appointments [67].

Current models that capture the impact of SDOH on HF care include the World Health Organization (WHO) and vulnerable population conceptual frameworks [66]. These models emphasize the interplay between available resources—such as community support, geographic factors, and individual-level healthcare access—and the significance of personal, social, and behavioral factors, including relative risk and the overall health status of HF patients. Based on these frameworks, vulnerable patients facing significant SDOH can benefit from socioeconomic and environmental resources within their communities, which influence health through individual lifestyles, cultural behaviors, and value systems [67]. These resources encompass a range of services, from pharmacies to social and rehabilitation services to telehealth [67].

Patients face real-world challenges when implementing lifestyle changes, particularly those with financial difficulties or limited access to healthcare resources. These challenges can negatively impact survival, quality of life, and readmission rates [67]. Individuals experiencing SDOH benefit from a dedicated team that facilitates their transition to post-acute care facilities, often through a transitional care coordinator. This team also helps improve access to prescribed medication regimens via various patient assistance programs or the Dispensary of Hope, a national charitable medication distributor [67].

However, uninsured patients may struggle to secure an appropriate outpatient follow-up for heart failure management. These individuals require resources to access care at local indigent clinics, federally funded clinics, or county hospitals as viable options for long-term care [67].

The economic burden of HF, like other diseases, is measured on many facets rooted in the financial burdens of direct and indirect costs [2]. Direct costs of HF include hospitalization, outpatient care, medications, rehabilitation, nursing care, and informal care [2]. Indirect costs are those associated with the total societal productivity loss based on standards such as ‘presenteeism’, or the lack of productivity from employees due to illness or disease even though they are physically present at work, sick leave, early retirement, and premature mortality [2]. In the PURE study, socioeconomic status (SES) and risk of cardiovascular disease in 20 low-income, middle-income, and high-income countries showed that hypertension, diabetes, and secondary prevention and care management were lower in people with the lowest levels of educational attainment among low-income countries [68]. However, less marked differences in cardiovascular health were found in healthcare disparities when comparing educational attainment among middle-income and high-income countries [68]. Possible explanations may include the allocation of budget spending on healthcare each fiscal year in various countries. Thus, low educational attainment in low-income countries equates to poor healthcare outcomes compared with high educational attainment in the same low-income country.

The inpatient cost of hospitalization for HF-related complications is by far the highest expenditure of all. The highest cost of hospitalization in any nation was noted in the US healthcare system, which approximated USD 125,000 per patient per year [69]. In most European countries, those hospitalization costs range from USD 5000 to as close as USD 18,000 per patient per year [69].

Economic disparities and increasing healthcare costs translate to immediate effects on compliance. Lower SES communities tend to present in later-stage HF due to avoidance of healthcare costs [70]. In an effort to address such disparities, risk calculators have begun incorporating factors related to SES, including zip codes within the US. With repeat and large-cost hospitalizations, patients accumulate a debt that results in difficulty optimizing care and future healthcare visits. This healthcare debt spiral leads to worsening HF outcomes [70].

In cases of low SES, lifestyle changes may be of utmost importance. Additional medical therapies that offer high efficacy and low cost should be considered. Initial medication therapy is crucial as patients are less likely to adhere to medication regimens if presented with initial high costs, even if subsequent therapies are more affordable [70]. Thus, we recommend opting for ACEi/ARB over ARNI and choosing metoprolol succinate before carvedilol in these patients. In a head-to-head trial of valsartan (ARB) to captopril (ACEi), ARB was shown to be non-inferior, but not superior, to ACEi [71]. As such, we suggest a selection of ACEi, reserving utilization of ARB if a patient demonstrates side effects of ACEi. Starting spironolactone as an earlier medication in GDMT may also be a consideration, with SGLT2i therapy used after confirming a patient can afford it.

## 5. Challenges and Considerations

The barriers to implementation and the challenges patients with HF face include both compliance and adherence to both non-pharmacological and pharmacological therapies. Generally speaking, patients who were more compliant with medications in various studies also adhered to effective lifestyle modifications, such as exercise, limited alcohol consumption, and smoking cessation, which all resulted in better outcomes [3].

The explanation of this cohesion among patients with HF and their successful adherence and compliance with non-pharmacological modifications also spills over to pharmacy-driven interventions. In one study, HF patients who were compliant with their non-pharmacological treatment regimen were more likely to also be compliant with medication and pharmacy interventions, which, when combined, led to a better prognosis for patients. Out of the four core non-pharmacological interventions mentioned prior (i.e., daily weighing, diet modification, management of fluid consumption, and exercise programs), compliance with an exercise program recommendation in the HF population was the lowest, nearing 60% [3].

Bridging the gap of noncompliance requires a multifaceted approach, particularly in addressing the underlying cause for the inability to adhere to their regimen [72]. Noncompliance rooted in medical illiteracy can be combated with aggressive education and social support. In contrast, financial noncompliance requires a more tailored approach to management that considers affordability and efficacy in therapies, as noted above.

Educational attainment plays a significant role in how many CRP sessions would be completed by the patient. Patients with at least a 4-year college degree were more likely to complete CR interventions than patients with either a high school or less than a high school/GED diploma with a *p* value of <0.001 [73].

## 6. Discussion and Future Directions

The ubiquity of HF affecting multiple organ systems and spanning different populations contributes to the complexity of HF management. Although a well-studied field, growing evidence regarding HF management continues to be a topic of discussion, both in management modalities and nuances in patient-centered care. Particularly, there are varying strengths of data in the management of HF in special populations, with more populations studied compared with others. Clinicians are left to create a personalized approach for each patient using their empiric experiences in combination with existing care algorithms. There appears to be a paucity of evidence-based data on management prioritization in the female population, marking an area of potential growth. Additionally, socioeconomic status proves to be a large consideration in HF management in practice, yet structured approaches to medication titration, compliance, and lifestyle education remain insufficient. Thus, managing such populations continues to be a challenge, warranting further research and guideline-directed approaches.

Broadly speaking, new methods for heart failure management continue to be evaluated, including targeted physical exercise, dietary benefits, and medications. Innovations in the field of HF have led to discoveries of targeted drug therapies intended for other diseases, which can now be generalized to the HF population. In turn, HF management can undergo a paradigm shift, with a transition toward a multisystem and holistic approach as opposed to managing HF as a separate entity. GLP-1 antagonists, in addition to SGLTi, are among some of the examples of emerging drugs as potential HF therapies. Nonselective medications are also a hot topic of research for classes of drugs within GDMT. With further investigation, such therapies may become incorporated into GDMT in the near future.

Interventional management of HF also serves as a new and innovative avenue for heart failure. CardioMems to guide medical therapy has been a relatively new approach. Though beyond the scope of this paper, therapies such as IVC occluders, interatrial septal shunting, renal denervation, and BaroStim are emerging and may grow in popularity for interventional approaches to HF management.

## 7. Conclusions

Heart failure is a complex syndrome with high morbidity and mortality, and it requires a multidisciplinary approach. Non-pharmacological therapies and heart failure management include patient education, lifestyle modifications, dietary changes, increased physical activity, psychosocial support, and specialized cardiac rehabilitation programs. A continuous educational program is vital for chronic heart failure patients, covering CHF causes, symptoms, diet, salt/fluid restrictions, medication adherence, and lifestyle adjustments. A multidisciplinary team of dietitians, physical therapists, psychologists, nurses, and social workers is recommended to improve patient well-being and reduce healthcare costs. These measures, when used in conjunction with medical therapy, provide a comprehensive approach to managing heart failure, empowering patients to actively participate in their care and achieve better health outcomes.

## Figures and Tables

**Table 1:** Key aspects of CRT and ICD therapies, including purpose, indications, long-term outcomes, and potential complications. Heart failure with reduced ejection fraction (HfrEF). Left ventricular ejection fraction (LVEF). Heart failure New York Heart Association (HF NYHA). QRS complex (QRS). Ventricular fibrillation (V-fib). Ventricular tachycardia (V-tach). Myocardial infarction (MI).

	Cardiac Resynchronization Therapy (CRT)	Implantable Cardioverter Defibrillator (ICD)
Purpose	Coordinates cardiac contractions, reduces mortality, prevents arrhythmias and sudden cardiac death (SCD)	Monitors heart rhythm, delivers shocks to prevent SCD
Indications	HfrEF with LVEF ≤ 35% Symptomatic HF NYHA class II, III, or ambulatory IV despite optimal therapy Prolonged QRS ≥ 130 ms	LVEF ≤ 35% Symptomatic HF History of V-fib or sustained V-tach Recent MI (within 40 days) with LVEF ≤ 35% and symptomatic HF Nonischemic cardiomyopathy with LVEF ≤ 35% Long-standing symptomatic HF despite optimal therapy
Long-term Outcomes	Lengthens time to heart transplantation and left ventricular assist device placement	Lower incidence of SCD in the ICD group, more pronounced in patients <70 years
Potential Complications	Perioperative complications (pneumothorax, bleeding, cardiac perforation)	Inappropriate shocks Device-related infection Fear of appropriate shocks Impact on quality of life

**Table 2:** HF management in special populations, including general principles and key considerations. Guideline-directed medical therapy (GDMT). Angiotensin-converting enzyme-inhibitor (ACEi). Angiotensin receptor blocker (ARB). Angiotensin receptor-neprilysin inhibitor (ARNI). Mineralocorticoid receptor antagonist (MRA). Heart failure (HF). Cardiac defibrillator therapy (CRT). Implantable cardioverter defibrillator (ICD). Chronic kidney disease (CKD). Sudden cardiac death (SCD). Estimated glomerular filtration rate (eGFR). Sodium-glucose cotransporter-2 inhibitor (SGLTi). Sodium-glucose cotransporter-2 (SGLT2). Glucagon-like peptide-1 (GLP-1). Dipeptidyl peptidase-4 (DPP-4).

Special Population	General Principle	Key Considerations
Elderly patients	Elderly patients with HF have similar GDMT recommendations as the general population but require tailored approaches due to increased sensitivity to medications and changes in intravascular volume.	Noncompliance: financial issues, social isolation, and cognitive impairments Medication sensitivity: requires nuanced medication selection ◦ Beta blockers and ACEi/ARBs/ARNIs are the most effective. ◦ MRAs are also beneficial. ◦ Diuretics effectively manage congestive symptoms. Exercise: improves muscle mass and strength Patient education and support: improve adherence and monitoring, potentially reducing disparity.
Female patients	Sex-specific research is limited, but certain lifestyle and physiological factors in women affect HF risk and management.	Diet: ◦ Plant-based diets are associated with a lower HF risk in women. ◦ Southern dietary patterns increase HF risk. Treat comorbidities: obesity, anemia, depression. Hormone therapy: estrogen therapy is generally not recommended due to the potential for thrombosis and worsening HF.
Patients with renal dysfunction	Both device-driven and pharmacological therapies are effective in managing HF with renal dysfunction, with specific interventions benefiting across different eGFR levels.	Device therapy: ◦ CRT: reduces mortality, HF hospitalizations, and incidence of VT. ◦ ICD: CKD patients have an increased risk of SCD. Pharmacological therapy: ◦ Standard GDMT medications: prioritize ACEi/ARB/ARNI and SGLT2i. ◦ Nonselective SGLTi: new data support use in CKD.
Patients with diabetes	Exercise and specific antihyperglycemic agents improve HF outcomes in patients with diabetes.	Exercise: ◦ Improves functional capacity and reverses cardiac stiffness. Antihyperglycemic agents ◦ SGLT2 inhibitors: improve cardiovascular outcomes (benefits seen even in patients without diabetes). ◦ GLP-1 agonists and DPP-4 inhibitors: improve weight loss, glycemic control, and cardiovascular health.
Ethnic and racial minority patients	Socioeconomic factors heavily influence HF outcomes, with financial barriers leading to later-stage HF presentation and reduced adherence.	Healthcare costs: HF-related hospitalizations are very costly, thus affecting compliance and HF outcomes. Medication accessibility: ◦ Cost-effective medication choices: consider ACEi/ARB over ARNI and metoprolol succinate over carvedilol to reduce upfront costs. ◦ Medication selection: spironolactone is suggested as an early GDMT medication, with SGLT2 inhibitors used after confirming affordability.

**Table 3:** The mechanisms, arterial changes, and hemodynamic consequences of aging in the cardiovascular system. Advanced glycation end products (AGEs). Left ventricular (LV). Matrix metalloproteases (MMPs).

Mechanisms	The mechanisms of aging, including increasing oxidative stress, endothelial dysfunction, chronic inflammation, activity of MMPs, and dysregulated vascular cell proliferation, collectively contribute to tissue damage, vascular aging, and the development of age-related diseases.
Changes in Arterial Wall	The changes in the arterial wall with aging include a decrease in elastin content, an increase in collagen production, cross-linking, and glycosylation, a decrease in collagen degradation, an increase in AGEs, and an increase in intima-medial thickness.
Hemodynamic Consequences	The hemodynamic consequences of aging include an increase in the diameter of central arteries, systolic pressure, pulse pressure, LV afterload, contraction, and oxygen requirements, along with a decrease in coronary filling pressure and LV early diastolic filling.

## Data Availability

No new data were created or analyzed in this study. Data sharing is not applicable to this article.
